# Novel Neonatal Variants of the Carbamoyl Phosphate Synthetase 1 Deficiency: Two Case Reports and Review of Literature

**DOI:** 10.3389/fgene.2019.00718

**Published:** 2019-08-22

**Authors:** Beibei Yan, Chao Wang, Kaihui Zhang, Haiyan Zhang, Min Gao, Yuqiang Lv, Xiaoying Li, Yi Liu, Zhongtao Gai

**Affiliations:** ^1^Neonatology Department, Qilu Children’s Hospital of Shandong University, Ji’nan, China; ^2^Shandong Freshwater Fisheries Research Institute, Ji’nan, China; ^3^Pediatric Research Institute, Qilu Children’s Hospital of Shandong University, Ji’nan, China

**Keywords:** carbamoyl phosphate synthetase 1 deficiency, carbamoyl phosphate synthetase 1, urea cycle disorders, next-generation sequencing, missense, nonsense, deletion, splicing

## Abstract

Carbamoyl phosphate synthetase I (CPS1) deficiency (CPS1D), is a rare autosomal recessive disorder, characterized by life-threatening hyperammonemia. In this study, we presented the detailed clinical features and genetic analysis of two patients with neonatal-onset CPS1D carrying two compound heterozygous variants of c.1631C > T (p.T544M)/c.1981G > T (p.G661C), and c.2896G > T (p.E966X)/c622-3C > G in *CPS1* gene, individually. Out of them, three variants are novel, unreported including a missense (c.1981G > T, p.G661C), a nonsense (c.2896G > T, p.E966X), and a splicing change of c.622-3C > G. We reviewed all available publications regarding *CPS1* mutations, and in total 264 different variants have been reported, with majority of 157 (59.5%) missense, followed by 35 (13.2%) small deletions. This study expanded the mutational spectrum of *CPS1*. Moreover, our cases and review further support the idea that most (≥90%) of the mutations were “private” and only ∼10% recurred in unrelated families.

## Introduction

Carbamoyl phosphate synthetase I (CPS1) deficiency (CPS1D) is a rare autosomal recessive urea cycle disorder, characterized by hyperammonemia with the incidence of 1/50,000 to 1/300,000 (Díez-Fernández et al., 2015). CPS1D is currently divided into two types of neonatal onset and late onset, whereas CPS1D with severe manifestations of hyperammonemia is common in neonatal-onset patients (Choi et al., 2017; Rokicki et al., 2017; Yang et al., 2017; Zhang et al., 2018). Typically, the neonatal-onset patient with CPS1D appears to be healthy at birth, but deteriorates rapidly into severe hyperammonemia, presenting poor feeding, vomiting, hypotonia, irritability, seizures, hypothermia, lethargy, coma, apnea, and even death after first feeding (Funghini et al., 2012; Choi et al., 2017; Rokicki et al., 2017; Zhang et al., 2018).

The function of urea cycle is to transform toxic ammonia into non-toxic urea. CPS1 catalyzes the initial and limiting step of the urea cycle, which is critical in the detoxification of excess ammonia, so CPS1D patient suffering from hyperammonemia will present a decreased level of citrulline but elevated glutamine in blood amino acid analysis, and a low level of orotic acid in urine test (Funghini et al., 2012; de Cima et al., 2015; Ali et al., 2016).

It is difficult to timely diagnose CPS1D due to atypical manifestations like sudden onset, rapid progress, and low morbidity, as well as complicated and non-recurrent genetic mutations in *CPS1* gene (Choi et al., 2017; Rokicki et al., 2017; Zhang and Li, 2017). For more than a decade, the diagnosis of CPS1D has been mainly relied on the laboratory tests of tandem mass spectrometry (MS/MS) including liquid chromatography-tandem mass spectrometry (LC-MS/MS) and gas chromatography mass spectrometry (GC/MS). MS/MS is a high-throughput technique for measurement of the intermediate metabolites and has been widely used to distinguish dozens of metabolic diseases (Lehotay et al., 2011; Janecková et al., 2012; Hao et al., 2018). However, this technology cannot differentiate CPS1D from *N*-acetylglutamate synthase deficiency (NAGSD) in UCDs due to their similar intermediate metabolites. Until recently, next-generation sequencing (NGS), a powerful DNA sequencing technology, has revolutionized genomic research with great utility in the molecular diagnosis of genetic disorders (Choi et al., 2017; Jia et al., 2017; Rokicki et al., 2017; Li et al., 2018; Zhang et al., 2018), and has been proven reliable and important to detect *CPS1* mutation for early diagnosis of CPS1D, as the severity of clinical manifestations in CPS1D patients is determined by the extent of CPS1 deficiency (Choi et al., 2017; Chen et al., 2018; Zhang et al., 2018).

In this study, we performed the clinical examinations and mutation analysis on two neonatal patients with CPS1D. The LC-MS/MS and GC/MS were carried out to detect amino acids in blood and organic acidurias in the urine, and then NGS was utilized to test the gene mutation. Strikingly, for the first time, we identified three novel pathogenic mutations of *CPS1*. To our knowledge, there have been so far only three reports of using NGS to detect *CPS1* mutations for CPS1D diagnosis (Choi et al., 2017; Chen et al., 2018; Zhang et al., 2018). Our novel findings further expanded the mutational spectrum of *CPS1* and demonstrated additional evidences of using NGS for precise identification of *CPS1* mutations in patients.

## Materials and Methods

### Patients, Samples and Ethical Approval

This study was approved by Medical Ethics Committee of Qilu Children’s Hospital of Shandong University. The written informed consents were obtained from the parents of each study participant, and the patients’ information was anonymized before submission. All the procedures performed in the study were in accordance with the Declaration of Helsinki.

Two patients from two unrelated families who were from the neonatal intensive care unit (NICU) of Qilu Children’s Hospital of Shandong University (QCHSU) were firstly screened by LC/MS-MS and GC/MS. The parents of both patients were healthy and non-consanguineous. Blood samples were obtained from the patients and their parents in accordance with informed consents in the study.

In addition, 100 blood samples from healthy children were collected as control samples for mutations validation.

### Routine Examination and Biochemical Laboratory Tests

Routine physical examination, complete blood count (CBC), C-reactive protein (CRP), hemoculture, and biochemical laboratory tests, such as liver function, kidney function, glucose, ammonia, lactic acid, and blood gas analyses, were carried out.

The level of orotic acids in urine was measured by GC/MS with GCMS-QP2010 analyzer (Shimadzu, Tokyo, Japan) and analyzed by the Inborn Errors of Metabolism Screening System software (Shimadzu), whereas amino acids level in blood was detected by LC-MS/MS with Applied Biosystems API 3200 analyzer (ABSCIEX, Foster City, CA) and analyzed by the ChemoView software (ABSCIEX).

### Next-Generation Sequencing and Variant Discovery

Genomic DNA was extracted and purified from peripheral blood of the two patients and their parents using TIANamp Blood Genomic DNA Purification Kit (Tiangen Biotech, Beijing, China). Whole-exome sequencing was applied to test mutation of genes in both patients. Approximately 3 µg of genomic DNA was randomly fragmented. An exome enriched kit (Agilent, Santa Clara, CA) was used to obtain the coding exons and flanking intronic regions. The sequencing was performed using HiSeq2000 sequencer (Illumina, San Diego, CA). The obtained mean exome coverage was over 99.2%, and average sequencing depth of each sample was 100%. Raw data obtained from the sequencer were further analyzed including read alignment, variant calling, and annotation by SinoPath Enterprise Ltd (Beijing, China). Low-quality reads (quality score ≤ 20 and sequencing depth ≤ 5) in the raw data were removed. Filtered reads were aligned to the human reference genome (UCSC hg19 Feb.2009) using the Burrows-Wheeler Aligner (Raney et al., 2014). Single-nucleotide variants (SNVs) and small insertions/deletions (indels) can be detected. Annotation was carried out by ANNOVAR for gene information, protein functional predictions, and population allele frequencies (Wang et al., 2010). Variants outside of coding regions and greater than 1% MAF (minor allele frequency) in the population were excluded.

### Bioinformatic Analysis and Verification of Mutations

All known variants were reported according to the following databases: OMIM (http://www.ncbi.nlm.nih.gov/omim/limits), UCSC Genome Bioinformatics (http://genome.ucsc.edu/), Human Gene Mutation Database (http://www.hgmd.cf.ac.uk/ac/index.php), Single Nucleotide Polymorphism Database (dbSNP) (http://www.ncbi.nlm.nih.gov/SNP/), 1000 Genomes Database (http://browser.1000genomes.org), ExAC (http://exac.broadinstitute.org/about), and gnomAD (http://gnomad.broadinstitute.org/). *In silico* analysis of the variants was carried out using PolyPhen-2 and SIFT and Mutation Taster to predict the pathogenicity. Human Splicing Finder (HSF) was applied to predict the effect of splicing variant. The multiple-sequence alignments were carried out by ClustalX. Modeling of affected protein structure was processed using SWISS-MODEL. The data analysis was conducted referring to the document (Jin et al., 2018). All the selected variants were classified as pathogenic, likely pathogenic, a variant of unknown significance (VUS), likely benign, or benign according to the American College of Medical Genetics and Genomics (ACMG) guidelines (Richards et al., 2015). The potential pathogenic mutations were validated by Sanger sequencing.

## Results

### Clinical Characteristics of Two Patients

The clinical manifestations and laboratory data from the two patients were summarized in **Table 1**. Both patients were neonatal-onset type presenting fulminant symptoms due to serious hyperammonemia so that the life-sustaining mechanical ventilation, medications of vasopressors, liquid infusion, and ammonia scavengers were administered.

**Table 1:** Clinical and laboratory data of the two patients with CPS1D.

Patients	P1	P2
**Gender**	Female	Female
**Age at onset**	2D	3D
**Age deceased**	5D	4D
**Complications during pregnancy**	Uneventful	uneventful
**Weeks gestation at delivery**	40	39^+5^
**Birth weight (kg), Apgar score**	2.95, 10	2.9, 10
**Clinical course**	fulminant	fulminant
**Clinical features**		
Fever	+	−
Seizure	+	−
Coma	+	+
Cyanosis	+	+
Breathing	weak	grunting
Hemorrhage	−	Lung, stomach
Cardiac failure	+	+
Poor feeding	+	+
Abdominal distention		+
Urine	oliguria	anuria
**Arterial blood gas analysis**		
PH (reference, 7.25-7.45)	7.13↓	7.10↓
PO_2_ (reference, 50-80 mm Hg)	67	72
PCO_2_ (reference, 40-60 mm Hg)	42	62↑
HCO_3_ (reference, 19-30 mmol/L)	12↓	19.2
BE (reference, –3 to +3)	−18↓	−10.5↓
**Blood routine test**		
Red blood cells (reference, 3.5-5.5×10^12^/L)	5.68↑	3.35↓
White blood cells (reference, 5.0-14.5×10^9^/L)	22.06↑	24.77
Platelet count (reference, 100-300×10^9^/L)	354↑	290
Hemoglobin (reference, 138-218 g/L)	195	117↓
**Urine routine test**		
BLD (negative)	++	+++
PRO (negative)	++	+
KET (negative)	–	–
**Blood biochemical tests**		
ALT (reference, 0-38 U/L)	37	24
Lactic acid (reference, 0.7-2.1 mmol/L)	5.8↑	5.6↑
Glucose (reference, 3.3-6.1 mmol/L)	0.3↓	12↑
Potassium (reference, 3.5-5.5 mmol/L)	7.4↑	4.6
Sodium (reference, 135-145 mmol/L)	154↑	142
AST (reference, 0-38 U/L)	130↑	78↑
CK (reference, 21-220 U/L)	936↑	780↑
CK-MB (reference, 0-25 U/L)	30↑	67↑
PCT (reference, ≤0.5 ng/ml)	67.322↑	0.794↑
ammonia (reference, 18-72 μmol/L)	1404↑	823↑
**Blood mass spectrometry profile**		
Citrulline (reference, 4-30 μmol/L)	3.82↓	3.08↓
Alanine (reference, 62.9-328 μmol/L)	1264.4↑	3337.99↑
Proline (reference, 72-293 μmol/L)	634.45↑	413.38↑
Ornithine (reference, 42-358 μmol/L)	69.01	106.95
**Urinary organic acids**		
Urinary orotic acid (reference, 0-2 mmol/L)	0	0
Urinary 3-MGA (reference, 0-4 mmol/L)	15.7↑	45.75↑
**Chest X-ray**		
Pneumonia	+	–
Pneumorrhagia	–	+
**Echocardiography**		
Ejection fraction	38%↓	37%↓
Patent ductus arteriosus	+	−
Patent foramen ovale	+	+
**CPS1 sequencing**		
Allele 1 (from father)	c.1631C > T (p.T544M)	c.2896G > T (p.E966X)
Allele 2 (from mother)	c.1981G > T (p.G661C)	c.622-3C > G

+positive, *↑*elevated, *↓*decreased.

Patient 1 (P1), a full-term female, the first child of healthy unrelated parents, was vaginally delivered. Her mother had regular prenatal care starting from 12 weeks of pregnancy. She was apparently healthy at birth with weight of 2.95 kg, Apgar score of 10 at 1 min and 5 min after birth. The following day, however, she had a fever, and then gradually developed hyporeactiveness presenting respiratory distress, seizures, and acute circulatory collapse so she was immediately transported to NICU in QCHSU from local hospital. Laboratory tests revealed the abnormal blood indexes of ammonia, 1,404 μmol/L (reference, 18–72 μmol/L); citrulline, 3.82 μmol/L (reference, 4–30 μmol/L); alanine, 1,264.4 μmol/L (reference, 62.9–328 μmol/L); lactic acid, 5.8 mmol/L (reference, 0.7–2.1 mmol/L); glucose, 0.3 mmol/L (reference, 3.3–6.1 mmol/L); and white blood cells 22.06×10^9^/L (reference, 5.0–14.5×10^9^/L), as well as abnormal urinary indexes of undetected orotic acid and elevated 3-MGA 15.7 mmol/L (ref. 0–4 mmol/L). The chest radiograph result reported pneumonia and possible atelectasis. Patient heart rate reached up to 180 beats/min but the ejection fraction was only 38% and no signs of congenital heart disease (**Table 1**).

The mechanical ventilation and medications of vasopressors, liquid infusion, and antibiotics, ammonia scavengers, such as dopamine, dobutamine, dilator, meropenem, lactulose, and l-arginine were administered immediately. Meanwhile, oral feeding was forbidden, and total parenteral nutrition with lower amino acid was administered. Unfortunately, the patient deteriorated continually into multiple-organ failure and even had cardiac arrest with no spontaneous breathing. Considering the poor prognosis, her parents gave up her treatment, and she died at age of 5 days.

Patient 2 (P2), a full-term girl, the second child of healthy unrelated parents, was vaginally delivered. The first child of the family died suddenly at the third day after birth without a definite diagnosis. Her mother had regular prenatal care, and she was normal at birth with a weight of 2.9 kg, Apgar score of 10 at 1 and 5 min after birth. On the third day, however, she had a sudden onset of hyperlactacidemia and deteriorated even faster than P1 did. At the beginning, she was hyporeactive presenting grunting and anorectic, but no manifestations of fever, vomiting, and seizures. Five hours later, she developed pneumorrhagia, gastrointestinal hemorrhage, and anuria, so that she was immediately transferred to NICU in QCHSU from her local hospital. On the way to the hospital, her heart rate and oxygen saturation could not be maintained; cardio-pulmonary resuscitation and mechanical ventilator had to be administered. Nevertheless, she deteriorated very quickly, presenting coma, shock, and irregular respirations. When she was admitted, she looked pale with reduced perfusion and a low ejection fraction (37.9%) in her echocardiography. The blood flowed out of her intratracheal tube and nose. Her pupil diameter was about 4 mm, and pupillary reflex disappeared. Laboratory tests revealed abnormal blood indexes of ammonia, 823 μmol/L; citrulline, 3.08 mmol/L; alanine, 3,337.99 μmol/L (reference, 62.9–328 μmol/L); lactic acid, 5.6 mmol/L; glucose, 12 mmol/L; and white blood cells, 24.77 × 10^9^/L, as well as abnormal urinary indexes of undetected orotic acid and increases 3-MGA 45.75 mmol/L. The chest radiograph showed exudative lesions, which matched her pulmonary hemorrhage.

This patient received immediate treatment that was similar as P1 with mechanical ventilation, vasopressors, liquid infusion, ammonia scavengers (such as lactulose and l-arginine), dopamine, dobutamine, dilator, as well as total parenteral nutrition with lower amino acid. She continued to deteriorate with tremendous speed and no sign of improvement after 13 h of admission and died at the age of 4 days.

### Genetic Analysis and Pathogenicity Prediction

**Figure 1:**
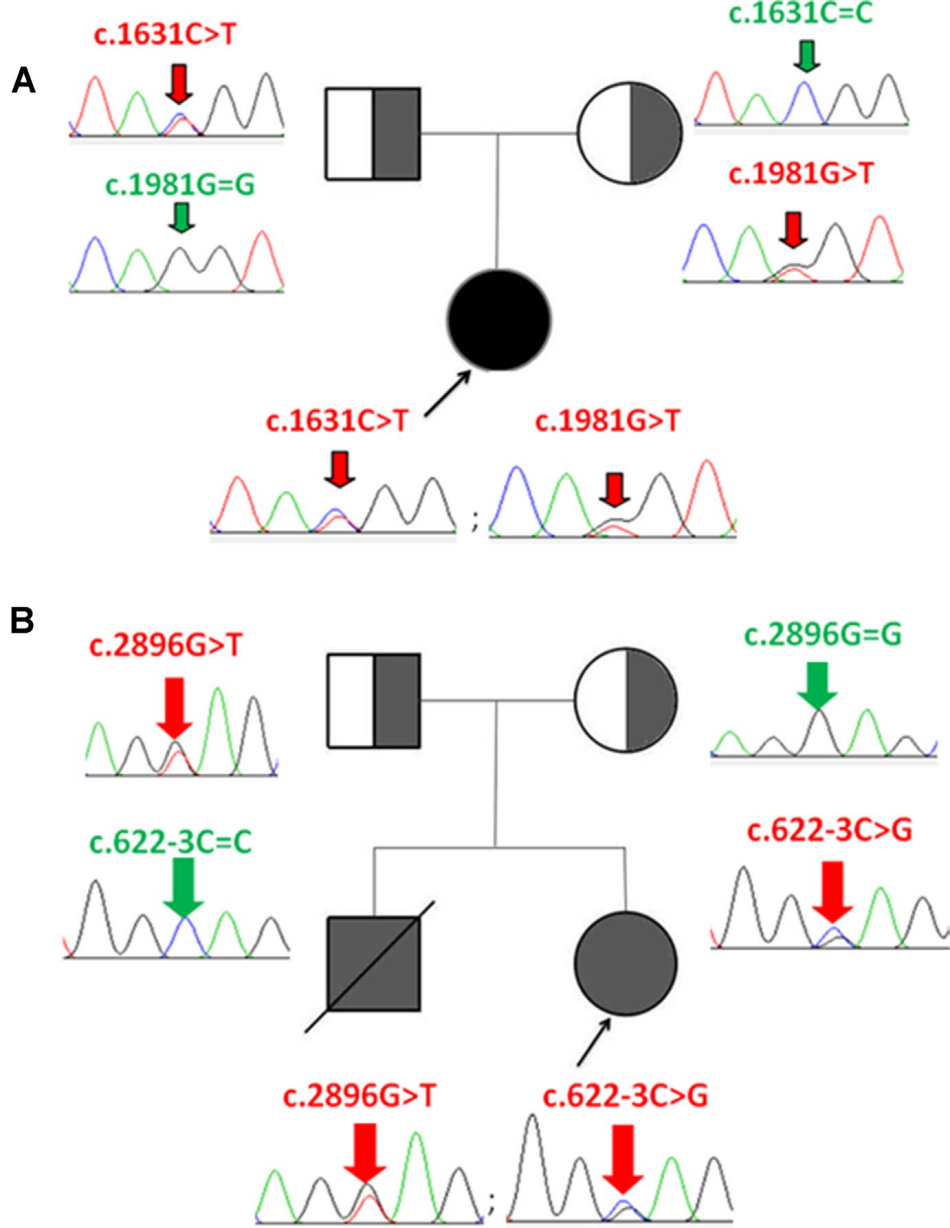
Identification of CPS1 mutations and pedigree of the two families with CPS1D probands. **(A)** P1 has two compound heterozygous mutations of c.1631C > T and c.1981G > T, inherited from her father and mother, respectively. **(B)** P2 has two compound heterozygous mutations of c.2896G > T and c.622-3C > G, inherited from her father and mother, respectively.

Whole-exome sequencing showed two compound heterozygous variants of the CPS1 gene in both P1 and P2, individually, including two missense variants of c.1631C > T (p.T544M) and c.1981G > T (p.G661C) found in P1, a nonsense variant c.2896G > T (p.E966X) and a splicing variant c.622-3C > G detected in P2. Of which, the variant c.1631C > T (p.T544M) was a known pathogenic mutation causing CPS1D (Finckh et al., 1998; Häberle et al., 2011) (**Table 1** and **Figures 1A, B**), whereas the remaining three variants of c.1981G > T (p.G661C), c.622-3C > G, and c.2896G > T (p.G966X) were novel and unreported in publications and public databases of OMIM, UCSC, HGMD, dbSNP, 1000 genomes, ExAC, and gnomAD. The missense c.1981G > T (p.G661C) occurred with an amino acid change from a nonpolar amino acid of glycine (G) to a polar amino acid of cysteine (C); the nonsense c.2896G > T (p.G966X) would create a premature stop codon; the splicing change c.622-3C > G was predicated to affect acceptor splice site. There were no mutations found in the control samples by using Sanger sequencing.

**Figure 2:**
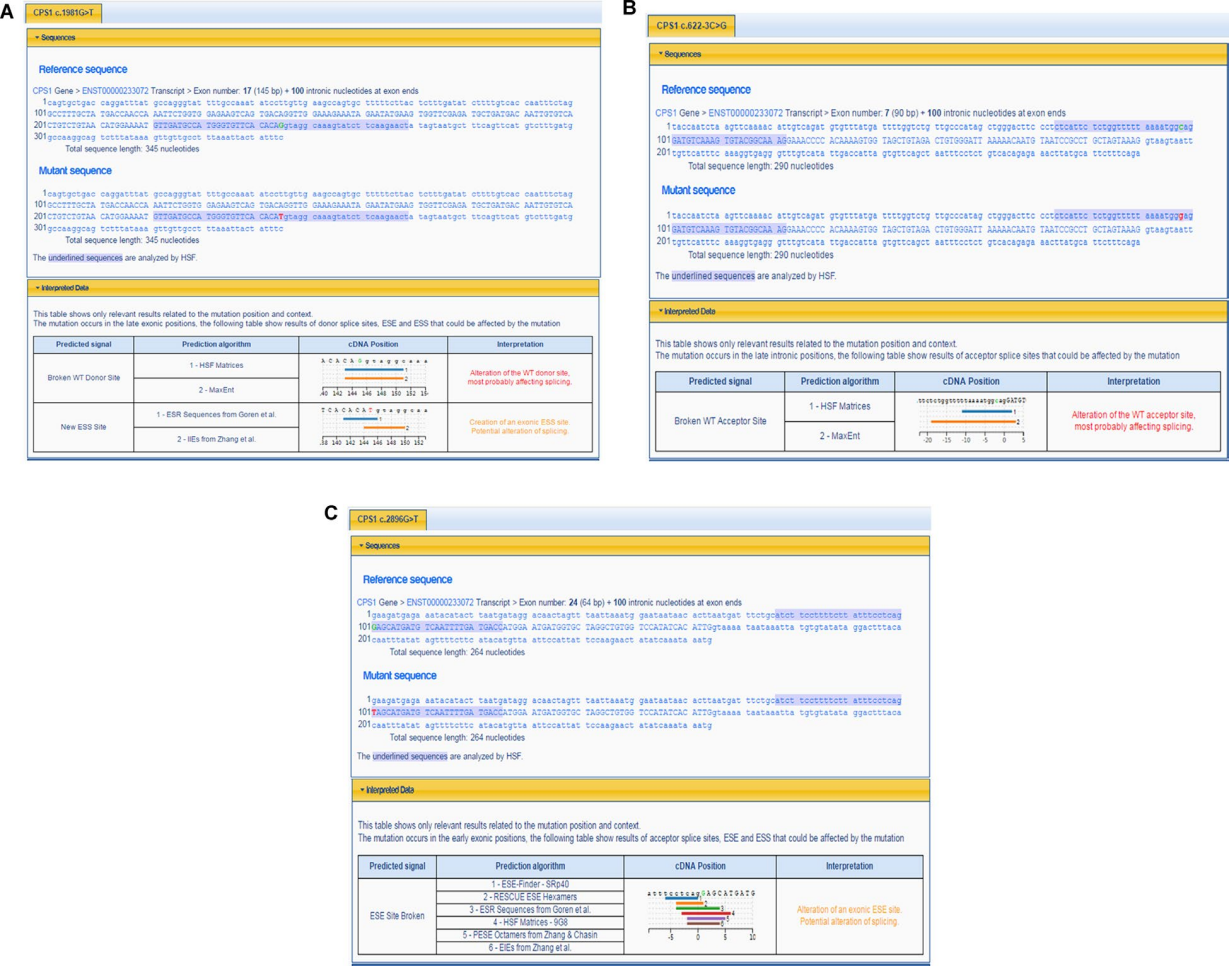
Prediction of splicing errors for three novel mutations of CPS1 by HSF. **(A)** Prediction of splicing errors for c.1981G > T of CPS1 shows either alteration of the donor site of *CPS1* affecting splicing or create an exonic ESS (exonic splicing silencer) site, potentially affecting splicing. **(B)** Prediction of splicing errors for c.622-3C > G of CPS1 shows the alteration of the acceptor site of *CPS1* affecting splicing. **(C)** Prediction of splicing errors for c.2896G > T of CPS1 shows alteration of an exonic ESE (exonic splicing enhancer) site, potentially affecting splicing.

**Figure 3:**
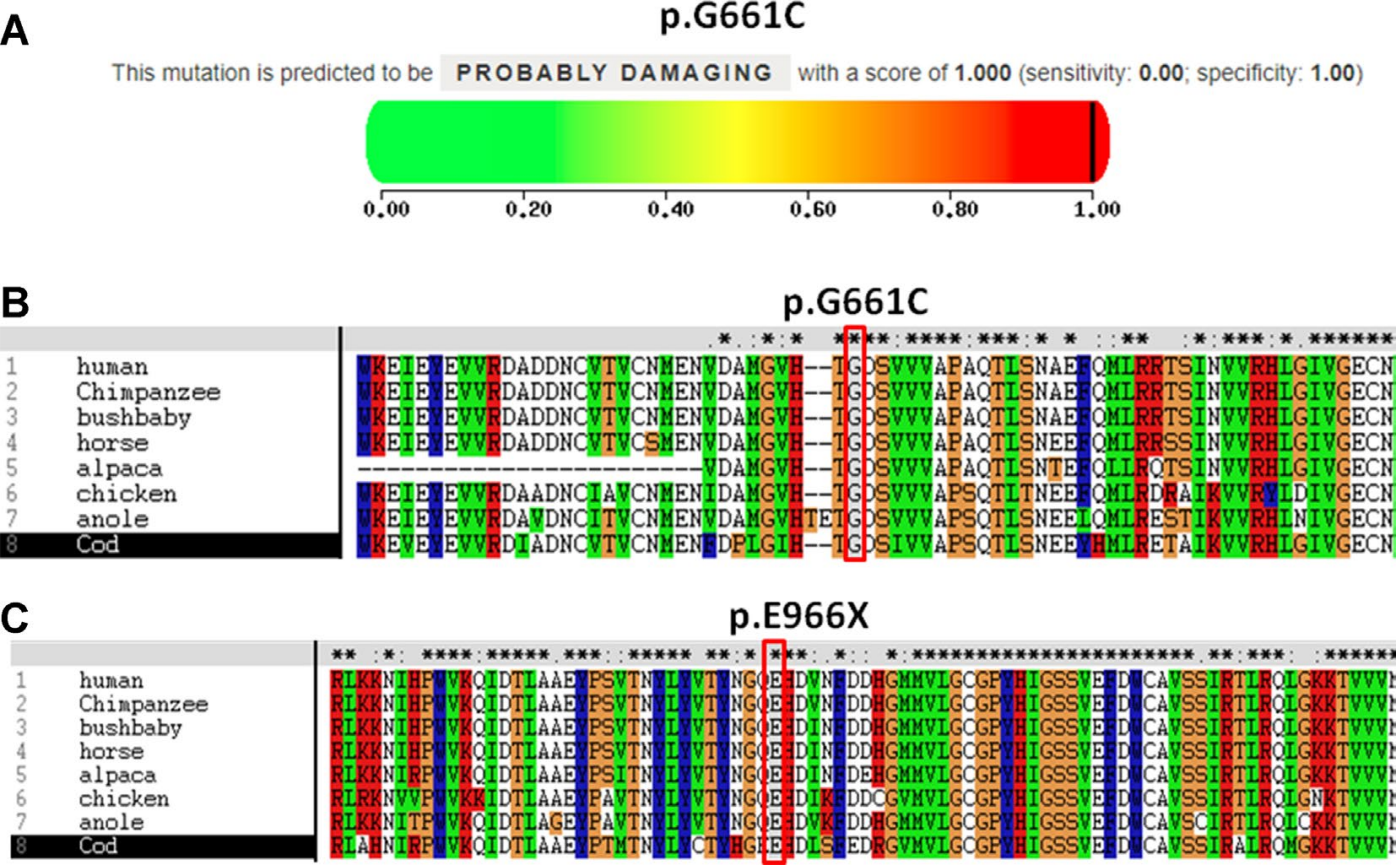
Pathogenicity and conservation analysis of the missense and nonsense mutations of CPS1. **(A)** Pathogenicity analysis of c.1981G > T (p.G661C) shows “probably damaging” with a score of 1.000 (sensitivity: 0.00; specificity: 1.00) by PolyPhen-2. **(B)** *In silico* analysis of c.1981G > T (p.G661C) in CPS1 shows the site p.G661 highly conservative in different species of human, chimpanzee, bushbaby, horse, alpaca, chicken, anole, and cod. **(C)** *In silico* analysis of c.2896G > T (p.E966X) in CPS1 shows the site p.E966 highly conservative in different species of human, chimpanzee, bushbaby, horse, alpaca, chicken, anole, and cod.

**Figure 4:**
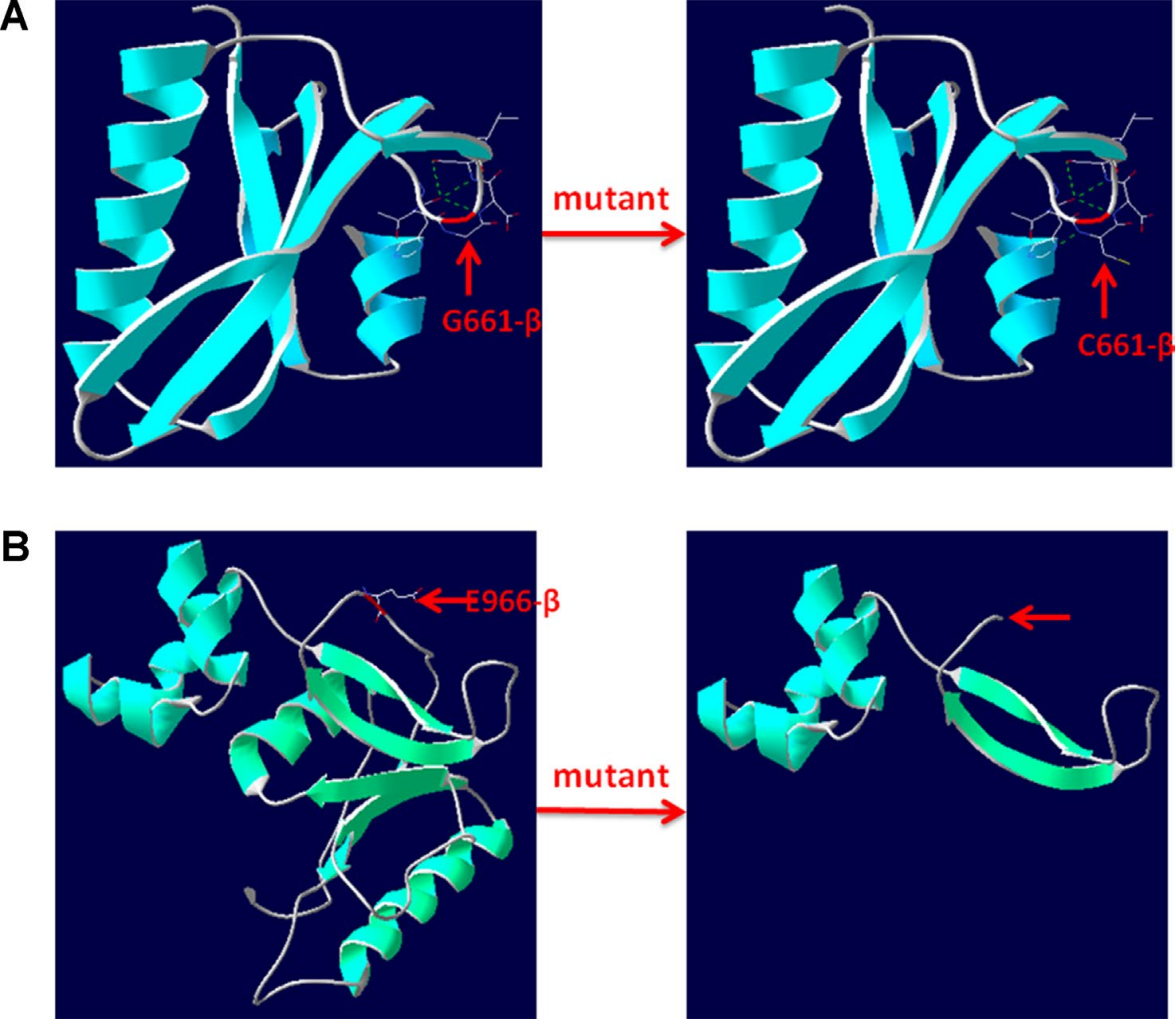
3D structure of wild type and mutant type of p.G661C and p.E966X in CPS1. **(A)** Wild type and mutant type of p.G661 in CPS1 shows the mutation of G661C changes the side strand structure and H-bond. **(B)** Wild type and mutant type of E966 in CPS1 shows the mutation of E661X leading to a truncated protein with missing 534 amino acids. The red arrows marked the sites of wild-type G661, E966, and mutated C661, X966.

The pathogenicity of three novel variants was further analyzed using various prediction online tools. In brief, HSF was applied to assess the potential impacts on the splicing of three novel variants as all these variants located near intron–exon junction. The predicted results showed that all three variants in the exon 17 (c.1981G > T), intron 7 (c.622-3C > G), and exon 24 (c.2896G > T) probably affect the splice sites (**Figures 2A–C**). The missense mutations of c.1981G > T (p.G661C) were predicted to be pathogenic by SIFT, MutationTaster, and PolyPhen-2 (**Figure 3A**). The conservation analysis of the variants of c.1981G > T (p.G661C) and c.2896G > T (p.G966X) in CPS1 showed that both sites were highly conservative in different species by using ClustalX (**Figures 3B, C**), whereas the missense variant c.1981G > T (p.G661C) was predicted to change the highly evolutionary conserved amino acid in CPS1, and the nonsense variant c.2896G > T (p.G966X) causing a premature stop could generate a truncated protein with missing conserved site of CPS1. In addition, the CPS1 protein crystallographic structure of both mutant types (p.G661C and p.G966X) revealed the changes of side strand structure and H-bond in variant of p.G661C, and a truncated protein with loss of 534 amino acids in variant of p.G966X (**Figures 4A, B**). All the mutation information and clinical data were uploaded into eRAM (Jia et al., 2018).

## Discussion

Pediatric rare diseases are often rapid deterioration with high mortality, which can be obviously improved by early diagnosis and treatment (Ni et al., 2017). CPS1D is a rare inborn error of UCD caused by CPS1 deficiency manifesting sudden onset, rapid progress, and low morbidity. In this study, we presented the detailed clinical manifestations and mutation analysis of two neonatal CPS1D cases. First, the blood ammonia, amino acids, and urine organic acids test results reported severe hyperammonemia on both patients, and the patients have very high levels of alanine and decreased levels of citrulline in blood, as well as the increased levels of 3-MGA and decreased levels of orotic acid in urine. We therefore referred patients for whole-exome sequencing to determine the genetic cause of this inborn error of metabolism. After validation of Sanger sequencing, two compound heterozygous variants in *CPS1* were identified in both patients, and one missense variant (c.1631C > T, p.T544M) was of known pathogenicity (Finchh et al., 1998; Häberle et al., 2011), whereas other three were novel and predicted to be pathogenic. Therefore, both patients were finally diagnosed as neonatal-onset CPS1D caused by *CPS1* mutations. To our knowledge, this study is the fifth case report of CPS1D in China and the 262–264th novel mutations in *CPS1* documented in the world (Chen et al., 2013; Yang et al., 2017; Chen et al., 2018; Zhang et al., 2018), which expands the mutation spectrum of *CPS1* (**Supplementary Tables S1** to **S4**).

CPS1 is an enzyme that catalyzes the first and rate-limiting reaction of three steps in ammonia detoxification of the urea cycle from ammonia to carbamoyl phosphate (de Cima et al., 2015; Ali et al., 2016). Normal function of the urea cycle requires six enzymes, including CPS1 as well as two mitochondrial transporters (Helman et al., 2014). CPS1 deficiency caused by *CPS1* gene mutation usually leads to accumulation of ammonia in the blood and thereby presents severe hyperammonemia, which is neurotoxic resulting in neonatal death or severe and irreversible brain damage in the developing and mature brain (Funghini et al., 2012; Choi et al., 2017; Rokicki et al., 2017; Yang et al., 2017). CPS1D are divided into two types of lethal neonatal-onset or less severe late-onset based on the age of onset, clinical features, and severity of CPS1 deficiency (Diez-Fernandez et al., 2017). To date, most of the reported CPS1D cases are neonatal-onset with severe hyperammonemia and usually died of multiorgan failure. Signs and symptoms in patients with CPS1D are often atypical with rapid progression and extremely low morbidity, which makes the clinical diagnosis difficult (Funghini et al., 2012; Choi et al., 2017; Rokicki et al., 2017).

As a result, the diagnosis of CPS1D is heavily dependent on laboratory data, such as blood ammonia, blood amino acids, urine organic acids, and genetic testing. The determination of blood ammonia concentration is critical for early clinical evaluation as it often reaches 150 μmol/L or higher in acute stage (László et al., 1991). Abnormal level of amino acids can be detected by mass spectrometry, like elevated blood glutamate, glutamine, and alanine, and reduced citrulline and arginine, whereas decreased urinary orotic acid and increased urinary 3-MGA (Lehotay et al., 2011; Janecková et al., 2012). Next-generation sequencing has been increasingly accessible in clinical laboratory for precise diagnosis of inborn errors of metabolism, including CPS1D (Choi et al., 2017; Yang et al., 2017; Li et al., 2018); for the biochemical tests mentioned above, one cannot distinguish from different types of UCD, particularly N-acetylglutamate synthase deficiency (NAGSD) from CPS1D due to similar intermediary metabolites (Choi et al., 2017).

*CPS1* (NM_001875.4) located on chromosome 2q34, spans over 122 kb consisting of 38 exons, which encode a polypeptide of 1,500 amino acids. Up to now, different variations of CPS1 have been reported, including missense, nonsense, small deletions, small insertions, small indels (insertions+deletions), and large deletions. As far as both cases of this study are concerned, two missense variants of c.1631C > T (p.T544M) and c.1981G > T (p.G661C) were found in a 2-day neonate girl (P1) with severe hyperammonemia. The variant of c.1631C > T (p.T544M) was a previously reported mutation (Finckh et al., 1998; Häberle et al., 2011) and was proven in the expression study causing large decrease of the enzyme activity due to hampering of the cross-talk between the bicarbonate phosphorylation domain (BPSD) and the allosteric NAG binding domain (ASD) (Diez-Fernandez et al., 2013). Another missense mutation (c.1981G > T, p.G661C) in P1 was novel and unreported. The pathogenicity of c.1981G > T (p.G661C) was analyzed and predicted as “deleterious” by SIFT, “protein features affected” by MutationTaster, and “probably damaging” with a score of 1.000 (sensitivity: 0.00; specificity: 1.00) by PolyPhen-2 (**Figure 3A**). As the variant occurred at the last exon base of the 17 exon, the potential splicing effect was assessed by HSF, which revealed a possible donor site error affecting the splicing of mRNA (**Figure 2A**). Moreover, the amino acid substitution site was highly conservative in different species analyzed by ClustalX (**Figure 3B**), so the alteration of p.G661C from glycine to cysteine was predicted to not only interrupt the conserved position of glycine but also change the side strand structure in the CPS1 protein crystallographic model, which produced a defective protein (**Figure 4A**). In addition, the mutation site of c.1981G > T (p.G661C) is identical as that of a known variant c.1981G > C (p.G661R) with the substitution of a small glycine residue by a large side chain, which was predicted to decrease structural stability of the protein (Funghini et al., 2012). Thus, we inferred that the substitution of amino acids from glycine to cysteine (p.G661C) might have a similar damage to the CPS1 stability, and both missense mutations in *CPS1*, leading to defects of enzyme function, are therefore the genetic cause of the patient with CPS1D.

A nonsense variant of c.2896G > T (p.G966X) and a splicing site change of c.622-3C > G were detected in a 3-day neonatal girl (P2). Since the nonsense variant site (c.2896G > T, p.G966X) is the first exon base of the 24th exon, its probable impact on splicing was assessed by HSF and revealed a potential effect of splicing with the alteration of an ESE (exonic splicing enhancer) site (**Figure 2C**). Besides, this position of glycine was highly conservative in different species analyzed by ClustalX (**Figure 3C**); the variant was predicted to generate a truncated protein with a loss of 534 amino acids and abolish the activity of the enzyme. The crystallographic structure model of the G966X further demonstrated the truncated protein (**Figure 4B**). The mutation of c.622-3C > G in intron 7 was a splicing site change that was predicted to alter the acceptor site of *CPS1* gene and affect mRNA splicing, which would produce a nonfunctional enzyme (**Figure 2B**). The severe phenotype of P2 with more rapid progress to multiple-organ failure within 13 h from her onset suggested that both alleles encode a nonfunctional protein. Additionally, an unusual death of the first boy in the family drew our attention. The first child was born at term and apparently healthy after a normal pregnancy. He had sudden deterioration and died on the third day without a definite diagnosis. The retrospective analysis of the first boy from this family demonstrated that he had similar features as P2. We conjectured that the first child might carry the identically compound heterozygous variants inherited from their father and mother as his sister P2. On this occasion, genetic counseling and prenatal genetic testing are necessary for the subsequent pregnancy. Considering the positions of the novel mutations and their potential splicing defects, RT-PCR should be used to identify the possibly aberrant transcripts; unfortunately, we failed to get the RNA from both patients due to their parents’ refusal.

**Figure 5:**
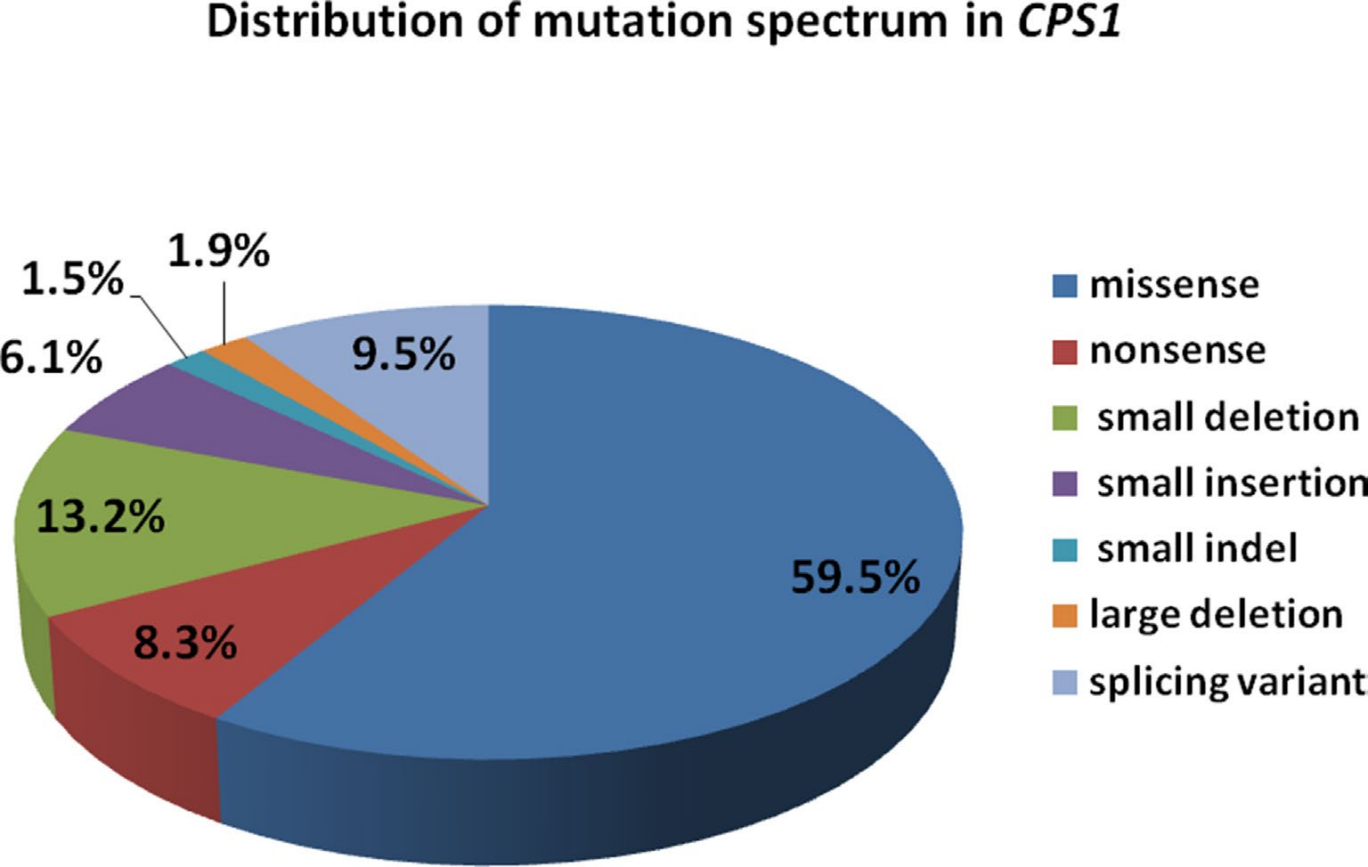
Distribution of mutation spectrum in CPS1 reported in both cases and literature. A total of 259 mutations in *CPS1* include 58.7% missense, 8.5% nonsense, 13.5% small deletion, 6.2% small insertion, 9.6% splicing, 1.6% small indel, and 1.9% large deletion.

We reviewed all publications of *CPS1* variants in cases of CPS1D, and a total of 264 *CPS1* different variations (including the 3 variants in this study) have been reported. Among them, the missense variants were the majority accounting for 157 (59.5%), followed by small deletions of 35 (13.2%), splice site changes of 25 (9.5%), and nonsense of 22 (8.3%), whereas the minority of the variants were 4 (1.5%) small indels and 5 (1.9%) large deletions with missing 1,000 bp to 767 kb, which were detected by genomic microarray (**Figure 5**). Of the variants, 81 (30.7%) were predicted to cause protein truncation, including 22 nonsense, 31 small deletions, 16 small insertions, 4 small indels, 6 splicing site changes, and 2 large deletions. Our reviewing data further clarified that most *CPS1* variants (≥90%) were “private” with non-recurrence, and the few recurrent mutations tended to occur at CpG dinucleotides, which made the diagnosis more complicated (**Supplementary Tables S1** to **S4**) (Hoshide et al., 1993; Finckh et al., 1998; Summar et al., 1998; Ihara et al., 1999; Aoshima et al., 2001a; Aoshima et al., 2001b; Rapp et al., 2001; Wakutani et al., 2001; Häberle et al., 2003; Eeds et al., 2006; Kurokawa et al., 2007; Khayat, 2009; Ono et al., 2009; Pekkala et al., 2010; Häberle et al., 2011; Wang et al., 2011; Funghini et al., 2012; Kretz et al., 2012; Diez-Fernandez et al., 2014; Ali et al., 2016; Choi et al., 2017; Rokicki et al., 2017; Yang et al., 2017; Chen et al., 2018; Zhang et al., 2018).

**Figure 6:**
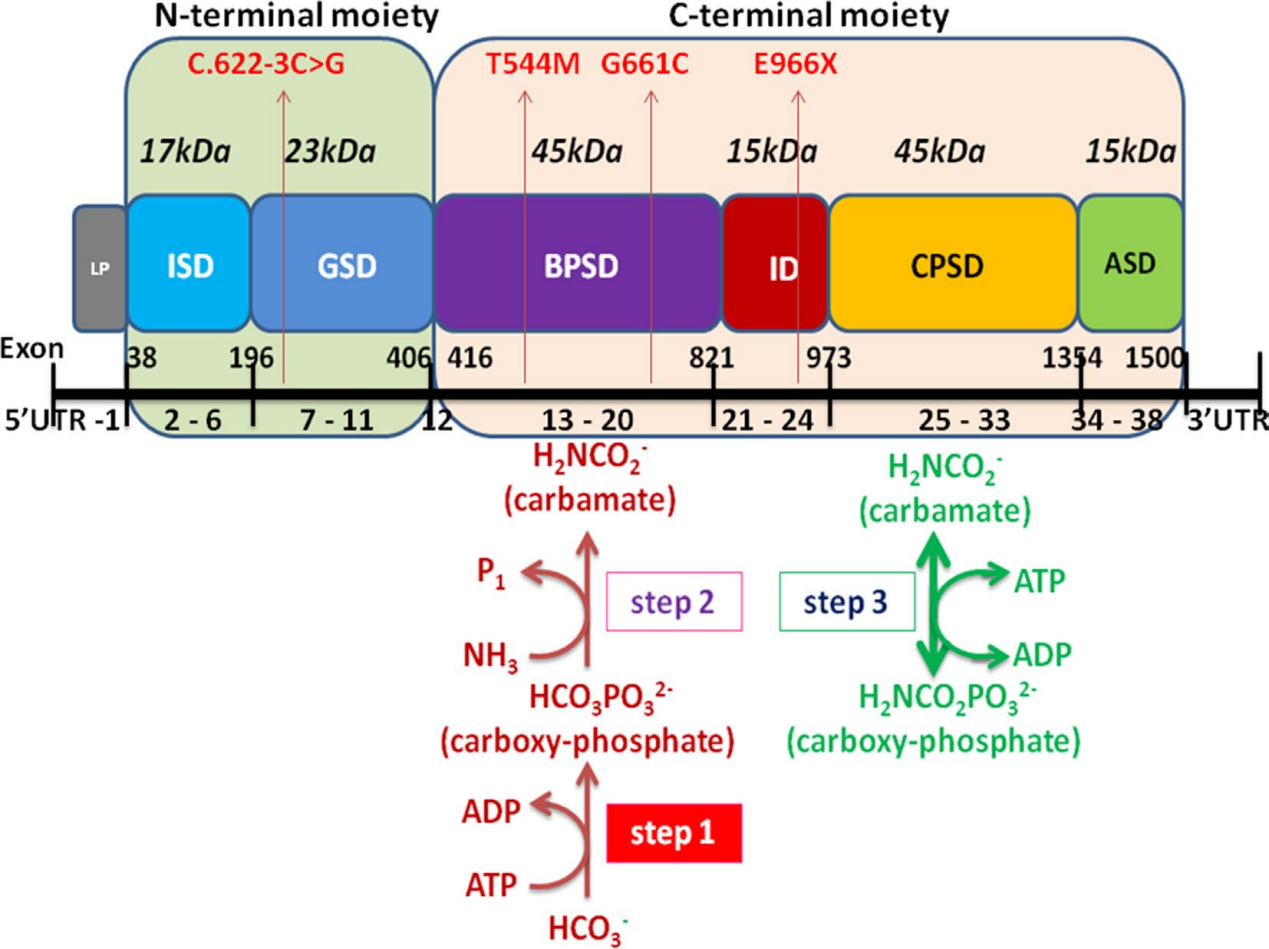
Schematic representation of the CPS1 domain and exons modified from Diez-Fernandez et al. (2014) and Ali et al. (2016). The CPS1 polypeptide consists of the 40-kDa N-terminal moiety and 120-kDa C-terminal human CPS1 domain that correspond to small and large subunits of *E. coli* CPS, respectively. The different color boxes represent the different domains of CPS1. LP mitochondrial targeting peptide is not present in mature CPS1. ISD, inter-subunit domain; GSD, ancestral inactive glutaminase; BPSD, bicarbonate phosphorylation; ID, integrating domain; CPSD, carbamate phosphorylation; ASD NAG binding domain. The black line at the bottom represents the exons of *CPS1*, including 5’UTR, exon 1-38, and 3′UTR. Four mutations of CPS1 in this study are shown in red arrow.

Encoded by *CPS1* gene, CPS1 is a complex multidomain enzyme, composed of a 40-kDa N-terminal moiety with two unknown function domains and a 120-kDa C-terminal moiety involving four domains of bicarbonate phosphorylation (BPSD), integrating (ID), carbamate phosphorylation (CPSD), and allosteric NAG binding (ASD) (Diez-Fernandez et al., 2014; de Cima et al., 2015). The C-terminal moiety contains two ATP-binding sites, catalyzing the synthesis of carbamoyl phosphate from bicarbonate, ATP, and ammonia and has been discovered to possess missense mutations of *CPS1* with high frequency and plays a critical integrating role in folding of structural elements leading to decreased yield of CPS1 (**Figure 6**) (Häberle et al., 2011; Diez-Fernandez et al., 2013; Diez-Fernandez et al., 2014). We analyzed the distribution of the 264 mutations and found that 66 (25%) variants were located at N-terminal moiety, whereas 198 (75%) mutations were at C-terminal moiety involving 76 variants at BPSD, 29 at ID, 75 at CPSD, and 18 at ASD. Three novel mutations were found in the study, two located at BPSD of C-terminal and one at N-terminal moiety, which further supported the importance of the C-terminal moiety in maintaining the function of CPS1.

At present, the treatment of CPS1D is to strictly follow the recommendations of UCDs, which focuses on reduction of ammonia production by a restricted protein diet and management of ammonia scavengers, such as sodium benzoate, sodium phenylbutyrate, and sodium phenylacetate, as well as drugs of l-arginine and l-citrulline to improve the residual urea cycle function and the renal excretion of ammonia (Diez-Fernandez and Häberle, 2017). In case of severe hyperammonemia, hemodialysis or peritoneal dialysis can be administered (Häberle, 2012; Diez-Fernandez and Häberle, 2017). However, these approaches cannot cure CPS1D, and the only available cure currently is liver transplantation, which has demonstrated excellent results with approximately 90% survival rate in UCD children, though it is limited by donor sources (Diez-Fernandez and Häberle, 2017; Zhang and Li, 2017). To date, most of the CPS1D patients died before receiving the confirmed diagnosis, so the detection of blood ammonia, blood amino acids, urine organic acid, and next-generation sequencing should be performed as early as possible.

## Conclusion

In this study, we presented the detailed clinical features and genetic analysis of two patients with neonatal-onset CPS1D and discovered three novel pathogenic variants in *CPS1* by whole-exome sequencing with a comprehensive outline of available publications regarding *CPS1* gene mutations. A total 264 different variants of *CPS1* have been reported with the majority of 157 (59.5%) missense, followed by small deletions of 35 (13.2%), and the minority of 5 (1.9%) large deletions and 4 (1.5%) indels, of which 81 (30.7%) were predicted to cause protein truncation. Our data further expand the spectrum of *CPS1* mutation and support the clinical applicability of whole-exome sequencing for genetic diagnosis of UCD.

## Ethics Statement

The work was approved by Medical Ethics Committee of Qilu Children’s Hospital of Shandong University. Written informed consents was obtained from the patients' parents and the patients’ information was anonymized before submission. All the procedures performed in the study were in accordance with the Declaration of Helsinki.

## Author Contributions

This study was conceived and designed by ZG and YL. The experiments were conducted by KZ, MG, YQL, and HZ. Data analyzed by KZ and YL. BY, XL and ZG contributed clinical diagnosis of the patients. The paper was written by BY, CW and YL.

## Conflict of Interest Statement

The authors declare that the research was conducted in the absence of any commercial or financial relationships that could be construed as a potential conflict of interest.
