# Supplementary material for: Novel Neonatal Variants of the Carbamoyl Phosphate Synthetase 1 Deficiency: Two Case Reports and Review of Literature
Source: Front Genet. 2019 Aug 22;10:718. doi: 10.3389/fgene.2019.00718 (PMC6713721; doi:10.3389/fgene.2019.00718)
Supplement: Supplementary file 1 [file Table_1.doc]

Table S1 Missense mutations reported in CPS1 gene

| **No** | **Exon** | **Nucleotide mutation** | **Protein** | **Reference** |
| --- | --- | --- | --- | --- |
| 1 | 2 | c.128C>T | p.A43V | Häberle et al., 2011 |
| 2 | 2 | c.173G>A | p.G58D | Häberle et al., 2011 |
| 3 | 2 | c.173G>T | p.G58V | Chen et al., 2018 |
| 4 | 2 | c.194C>T | p.S65F | Häberle et al., 2011 |
| 5 | 2 | c.212T>G | p.V71G | Häberle et al., 2011 |
| 6 | 2 | c.236G>A | p.G79E | Kurokawa et al.,2007 |
| 7 | 3 | c.259C>T | p.P875S | Häberle et al., 2011 |
| 8 | 3 | c.265T>G | p.Y89D | Häberle et al., 2011 |
| 9 | 3 | c.296C>T | p.P99L | Kretz et al., 2012 |
| 10 | 3 | c.368C>T | p.S123F | Summar et al., 1998 |
| 11 | 3 | c.368C>A | p.S123T | Ali et al., 2016 |
| 12 | 4 | c.323G>A | p.G108E | Yang et al., 2017 |
| 13 | 4 | c.446T>C | p.Leu149Ser | Chen et al., 2017 |
| 14 | 5 | c.494A>G | p.D165G | Häberle et al., 2011 |
| 15 | 5 | c.520C>T | p.R174W | Ali et al., 2016 |
| 16 | 7 | c.634T>A | p.Y212N | Kurokawa et al., 2007 |
| 17 | 7 | c.671A>T | p.D224V | Häberle et al., 2011 |
| 18 | 7 | c.697C>T | p.R233C | Häberle et al., 2011 |
| 19 | 8 | c.728A>C | p.H243P | Häberle et al., 2011 |
| 20 | 8 | c.773G>A | p.G258E | Häberle et al., 2011 |
| 21 | 8 | c.788G>A | p.G263E | Häberle et al., 2011 |
| 22 | 8 | c.794C>T | p.P265L | Kretz et al.,2012 |
| 23 | 8 | c.796G>A | p.G266R | chen et al., 2018 |
| 24 | 8 | c.840G>C | p.K280N | Kurokawa et al.,2007 |
| 25 | 9 | c.902G>A | p.G301E | Eeds et al.,2006 |
| 26 | 9 | c.911C>T | p.A304V | Häberle et al., 2011 |
| 27 | 10 | c.950G>A | p.G317E | Häberle et al., 2011 |
| 28 | 10 | c.1010A>G | p.H337R | Aoshima et al., 2001 |
| 29 |  | c.1022 T>C | p.L341S | Funghini et al.,2012 |
| 30 | 10 | c.1063A>G | p.N355D | Eeds et al., 2006 |
| 31 | 10 | c.1072G>C | p.D358H | Häberle et al., 2011 |
| 32 | 11 | c.1145C>T | p.P382L | Häberle et al., 2011 |
| 33 | 12 | c.1166A>G | p.Y389C | Eeds et al., 2006 |
| 34 | 12 | c.1169T>G | p.L390R | Eeds et al., 2006 |
| 35 | 12 | c.1201G>C | p.G401R | Häberle et al., 2011 |
| 36 | 13 | c.1291G>A | p.G431R | Häberle et al., 2011 |
| 37 | 13 | c.1295G>T | p.G432V | Häberle et al., 2011 |
| 38 | 13 | c.1312G>A | p.A438T | Häberle et al., 2011 |
| 39 | 13 | c.1312G>C | p.A438P | Kurokawa et al., 2007 |
| 40 | 13 | c.1348A>G | p.K450E | Häberle et al., 2011 |
| 41 | 14 | c.1370T>G | p.V457G | Funghini et al., 2003 |
| 42 | 14 | c.1412C>A | p.T471N | Pekkala et al., 2010 |
| 43 | 14 | c.1492G>C | p.A498P | Häberle et al., 2011 |
| 44 | 15 | c.1592T>A | p.V531E | Häberle et al., 2011 |
| 45 | 15 | c.1592T>G | p.V531G | Häberle et al., 2011 |
| 46 | 15 | c.1631C>T | p.T544M | Finckh et al.,1998; Häberle et al., 2011; This study |
| 47 | 16 | c.1759C>T | p.R587C | Häberle et al., 2011 |
| 48 | 16 | c.1760G>A | p.R587H | Kurokawa et al., 2007; Wang et al., 2011; Häberle et al., 2011 |
| 49 | 16 | c.1760G>T | p.R587L | Häberle et al., 2011 |
| 50 | 16 | c.1765G>A | p.A589T | Eeds et al., 2006 |
| 51 | 16 | c.1777G>C | p.G593R | Kurokawa et al., 2007 |
| 52 | 16 | c.1790C>T | p.S597L | Häberle et al., 2011 |
| 53 |  | c.1799G>A | p.C600Y | Zhang et al.,2018 |
| 54 | 17 | c.1864G>A | p.V622M | Häberle et al., 2011 |
| 55 | 17 | c.1883G>A | p.G628D | Häberle et al., 2011 |
| 56 | 17 | c.1895T>G | p.I632R | Häberle et al., 2011 |
| 57 | 17 | c.1913G>C | p.R638P | Häberle et al., 2011 |
| 58 | 17 | c.1918G>T | p.A640S | Eeds et al., 2006 |
| 59 | 17 | c.1943G>A | p.C648Y | Häberle et al., 2011 |
| 60 | 17 | c.1951G>A | p.E651K | Kurokawa et al., 2007 |
| 61 | 17 | c.1961A>T | p.D654V | Häberle et al., 2011 |
| 62 | 17 | c.1981G>C | p.G661R | Funghini et al., 2012 |
| 63 | 17 | c.1981G>T | p.G661C | This study |
| 64 | 18 | c.2021A>T | p.N674I | Kurokawa et al., 2007 |
| 65 | 18 | c.2022T>A | p.N674K | Häberle et al., 2011 |
| 66 | 18 | c.2033A>C | p.Q678P | Pekkala et al., 2010 |
| 67 | 18 | c.2093A>G | p.N698S | Häberle et al., 2011 |
| 68 | 18 | c.2148T>A | p.N716K | Summar et al., 1998; Eeds et al., 2006 |
| 69 | 18 | c.2153G>A | p.R718K | Häberle et al., 2011 |
| 70 | 18 | c.2162G>A | p.R721Q | Häberle et al., 2011 |
| 71 | 18 | c.2170G>C | p.A724P | Häberle et al., 2011 |
| 72 | 18 | c.2176G>A | p.A726T | Häberle et al., 2011 |
| 73 | 19 | c.2300A>T | p.D767V | Häberle et al., 2011 |
| 74 | 19 | c.2321C>T | p.P774L | Pekkala et al. [2010] |
| 75 | 19 | c.2339G>A | p.R780H | Kurokawa et al., 2007; Häberle et al., 2011 |
| 76 | 19 | c.2376G>C | p.M792I | Häberle et al., 2011 |
| 77 | 20 | c.2407C>A | p.R803S | Häberle et al., 2011 |
| 78 | 20 | c.2407C>G | p.R803G | Ono et al.,2009; Häberle et al., 2011 |
| 79 | 20 | c.2407C>T | pR803C | Häberle et al., 2011 |
| 80 | 20 | c.2413T>C | p.F805L | Eeds et al., 2006 |
| 81 | 20 | c.2414T>C | p.F805S | Häberle et al., 2011 |
| 82 | 20 | c.2429G>A | p.Q810R | Funghini et al., 2003 & Eeds et al., 2006 |
| 83 | 20 | c.2440C>T | p.R814W | Rokicki et al., 2017; Häberle et al., 2011 |
| 84 | 20 | c.2446T>C | p.C816R | Häberle et al., 2011 |
| 85 | 20 | c.2528T>C | p.L843S | Häberle et al., 2003 |
| 86 | 20 | c.2537C>T | p.P846L | Zhang et al., 2018 |
| 87 | 20 | c.2548C>T | p.R850C | Kurokawa et al., 2007 |
| 88 | 20 | c.2549G>A | p.R850H | Wakutani et al., 2004; Häberle et al., 2011 |
| 89 | 21 | c.2611A>C | p.T871P | Kretz et al., 2012 |
| 90 | 21 | c.2623A>G | p.K875E | Häberle et al., 2003 |
| 91 | 22 | c.2732G>A | p.G911E | Häberle et al., 2011 |
| 92 | 22 | c.2732G>T | p.G911V | Eeds et al., 2006 |
| 93 | 22 | c.2738C>T | p.S913L | Häberle et al., 2011 |
| 94 | 22 | c.2740G>C | p.D914H | Häberle et al., 2011 |
| 95 | 22 | c.2741A>G | p.D914G | Häberle et al., 2011 |
| 96 | 22 | c.2752T>C | p.S918P | Wakutani et al., 2004 |
| 97 | 22 | c.2795G>C | p.R932T | Häberle et al., 2011 |
| 98 | 23 | c.2845G>A | p.A949T | Häberle et al., 2011 |
| 99 | 23 | c.2873T>C | p.L958P | Eeds et al., 2006 |
| 100 | 23 | c.2876A>G | p.Y959C | Häberle et al., 2011 |
| 101 | 23 | c.2885A>G | p.Y962C | Häberle et al., 2011 |
| 102 | 23 | c.2891G>A | p.G964D | Funghini et al., 2012 |
| 103 | 24 | c.2933T>A | p.V978E | Häberle et al., 2011 |
| 104 | 24 | c.2944G>A | p.G982S | Eeds et al., 2006 |
| 105 | 24 | c.2945G>A | p.G982D | Kurokawa et al., 2007; Häberle et al., 2011 |
| 106 | 24 | c.2945G>T | p.G982V | Wang et al., 2011; Häberle et al., 2011 |
| 107 | 24 | c.2950T>C | p.Y984H | Häberle et al., 2011 |
| 108 | 24 | c.2957T>C | p.I986T | Häberle et al., 2011 |
| 109 | 24 | c.2959G>T | p.G987C | Häberle et al., 2011 |
| 110 | 25 | c.2975T>C | p.F992S | Häberle et al., 2011 |
| 111 | 25 | c.2993C>T | p.S998F | Eeds et al., 2006 |
| 112 | 25 | c.3047A>G | p.N1016S | Häberle et al., 2011 |
| 113 | 25 | c.3050C>T | p.P1017L | Häberle et al., 2011 |
| 114 | 25 | c.3065C>T | p.T1022I | Häberle et al., 2011 |
| 115 | 25 | c.3101A>G | p.E1034G | Häberle et al., 2011 |
| 116 | 25 | c.3134A>G | p.H1045R | Häberle et al., 2011 |
| 117 | 26 | c.3161T>G | p.I1054R | Rapp et al., 2001 |
| 118 | 26 | c.3176A>G | p.Q1059R | Häberle et al., 2011 |
| 119 | 26 | c.3194C>A | p.A1065E | Häberle et al., 2011 |
| 120 | 26 | c.3265C>T | p.R1089C | Khayat et al., 2009; Häberle et al., 2011 |
| 121 | 26 | c.3266G>T | p.R1089L | Summar et al.,1998; Eeds et al., 2006 |
| 122 | 26 | c.3308A>G | p.Q1103R | Kurokawa et al., 2007 |
| 123 | 28 | c.3422T>G | p.V1141G | Wakutani et al., 2004; Kurokawa et al., 2007 |
| 124 | 28 | c.3443T>A | p.M1148K | Zhang et al., 2018 |
| 125 | 28 | c.3464C>A | p.A1155E | Häberle et al., 2011 |
| 126 | 28 | c.3464C>T | p.A1155V | Häberle et al., 2011 |
| 127 | 29 | c.3500C>G | p.T1167R | Funghini et al., 2012 |
| 128 | 30 | c.3582A>C | p.E1194D | Kretz et al., 2012 |
| 129 | 30 | c.3584A>C | p.H1195P | Kurokawa et al., 2007 |
| 130 | 30 | c.3607T>C | p.S1203P | Summar et al., 1998; Eeds et al., 2006 |
| 131 | 30 | c.3608C>T | p.S1203L | Häberle et al., 2011 |
| 132 | 30 | c.3613G>A | p.D1205N | Eeds et al., 2006 |
| 133 | 30 | c.3643A>G | p.I1215V | Kurokawa et al., 2007 |
| 134 | 31 | c.3683G>A | p.R1228Q | Häberle et al., 2011 |
| 135 | 31 | c.3691G>C | p.A1231P | Rokicki et al., 2017 |
| 136 | 31 | c.3723C>A | p.N1241K | Kurokawa et al., 2007 |
| 137 | 32 | c.3760A>T | p.I1254F | Ali et al., 2016 |
| 138 | 32 | c.3765G>C | p.E1255D | Häberle et al., 2011 |
| 139 | 32 | c.3785G>A | p.R1262Q | Häberle et al., 2011 |
| 140 | 32 | c.3785G>C | p.R1262P | Häberle et al., 2011 |
| 141 | 32 | c.3820G>C | p.D1274H | Häberle et al., 2011 |
| 142 | 33 | c.3979T>C | p.C1327R | Häberle et al., 2011 |
| 143 | 33 | c.3980G>A | p.C1327Y | Rokicki et al., 2017 |
| 144 | 33 | c.3991T>C | p.S1331P | Eeds et al., 2006 |
| 145 | 33 | c.3998G>A | p.G1333E | Häberle et al., 2011 |
| 146 | 35 | c.4112G>T | p.R1371L | Häberle et al., 2011 |
| 147 | 35 | c.4132G>A | p.A1378T | Eeds et al., 2006 |
| 148 | 35 | c.4142T>C | p.L1381S | Summar et al., 1998 |
| 149 | 36 | c.4172C>T | p.T1391M | Häberle et al., 2011 |
| 150 | 36 | c.4192C>G | p.L1398V | Häberle et al., 2011 |
| 151 | 36 | c.4232C>T | p.P1411L | Summar et al., 1998; Eeds et al., 2006 |
| 152 | 37 | c.4316C>T | p.P1439L | Häberle et al., 2011 |
| 153 | 37 | c.4327A>G | p.T1443A | Eeds et al., 2006 |
| 154 | 37 | c.4357C>T | p.R1453W | Häberle et al., 2011; Pekkala et al., 2010 |
| 155 | 37 | c.4358G>A | p.R1453Q | Pekkala et al. , 2010 |
| 156 | 37 | c.4385C>G | p.P1462R | Häberle et al., 2011 |
| 157 | 38 | c.4471T>C | p.Y1491H | Summar et al., 1998 |

**Table S2** Nonsense mutations in the CPS1 gene

| **No** | **Exon** | **Nucleotide mutation** | **Protein** | **Reference** |
| --- | --- | --- | --- | --- |
| 158 | 2 | c.130C>T | p.Q44X | Ihara et al., 1999; Kurokawa et al., 2007 |
| 159 | 3 | c.267C>G | p.Y89X | Häberle et al., 2011 |
| 160 | 4 | c.420C>A | p.Y140X | Eeds et al., 2006 |
| 161 | 6 | c.580C>T | p.Q194X | Choi R et al., 2017 |
| 162 | 8 | c.712C>T | p.R238X | Häberle et al., 2003 |
| 163 | 11 | c.1123C>T | p.Q375X | Aoshima et al., 2001b |
| 164 | 13 | c.1289C>G | p.S430X | Rokicki et al., 2017 |
| 165 | 14 | c.1432C>T | p.Q478X | Eeds et al., 2006 |
| 166 | 15 | c.1615G>T | p.E539X | Häberle et al., 2011 |
| 167 | 15 | c.1642C>T | p.Q548X | Ali et al., 2016 |
| 168 | 16 | c.1770T>G | p.Y590X | Häberle et al., 2011 |
| 169 | 18 | c.2161C>T | p.R721X | Häberle et al., 2003 |
| 170 | 19 | c.2359C>T | p.R787X | Rapp et al., 2001; Kurokawa et al., 2007 |
| 171 | 23 | c.2893C>T | p.Q965X | Häberle et al., 2011 |
| 172 | 24 | c.2896G>T | p.E966X | This study |
| 173 | 25 | c.3093C>A | p.Y1031X | Kurokawa et al., 2007 |
| 174 | 25 | c.3136C>T | p.Q1046X | Rokicki et al., 2017 |
| 175 | 26 | c.3317G>A | p.W1106X | Häberle et al., 2011 |
| 176 | 29 | c.3520C>T | p.R1174X | Häberle et al., 2011 |
| 177 | 32 | c.3784C>T | p.R1262X | Wakutani et al., 2004; Eeds et al., 2006; Kurokawa et al., 2007 ; Ono et al., 2009 |
| 178 | 33 | c.3953T>A | p.L1318X | Funghini et al., 2012 |
| 179 | 36 | c.4229G>A | p.W1410X | Kretz et al., 2012 |

**Table S3 Deletions, insertions and splice errors of the CPS1 gene**

causing enzyme truncation

| **No** | **Exon** | **Nucleotide mutation** | **Protein** | **Reference** |
| --- | --- | --- | --- | --- |
| 180 | 1 | c.125delA | p.K42RfsX15 | Häberle et al., 2011 |
| 181 | 2 | c.154_160delGATGGAA | p.D52LfsX3 | Häberle et al., 2011 |
| 182 | Intron 2/Exon 3-Exon 38 | Intron 2_3’UTRdel134kb_767kb(Intron 2_3’UTRdelExon3-38) | No exact breakpoints available | Wang et al., 2011 |
| 183 | 4 | c.409_410insTATA | p.S137IfsX2 | Häberle et al., 2011 |
| 184 | 5 | c.527_528insGA | p.G177RfsX25 | Häberle et al., 2011 |
| 185 | 7 | c.700delC | p.L234CfsX2 | Häberle et al., 2011 |
| 186 | 8 | c.731delT | p.L244X | Eeds et al., 2006 |
| 187 | 9 | c.848delA | p.E283GfsX16 | Häberle et al., 2011 |
| 188 | 9 | c.850delA | p.S284VfsX15 | Rokicki et al., 2017 |
| 189 | 9 | c.860delA | p.K287RfsX12 | Kurokawa et al., 2007 |
| 190 | 9 | c.866delC | p.P289HfsX10 | Häberle et al., 2011 |
| 191 | 9 | c.900_901insAG | p.G301EfsX24 | Häberle et al., 2011 |
| 192 | 12 | c.1195_1196delAA | p.K399EfsX22 | Eeds et al., 2006 |
| 193 | 14 | c.1390_1397delCCAAACAT | p.P464CfsX7 | Häberle et al., 2011 |
| 194 | 14 | c.1413_1414insC | p.N472QfsX2 | Häberle et al., 2011 |
| 195 | 14 | c.1440_1441delTA | p.Thr481CysfsX58 | Funghini et al., 2012 |
| 196 | 14 | c.1528delG | p.G510AfsX5 | Wakutani et al., 2004; Kurokawa et al., 2007 |
| 197 | 14 | c.1547delG | p.G516AfsX5 | Kurokawa et al., 2007 |
| 198 | Intron14 | c.1549+1G>T (c.1420_1549del130) | p.V474EfsX15  Donor splice site error | Häberle et al., 2011 |
| 199 | 16 | c.1778insG | p.G594WfsX42 | Kretz et al., 2012 |
| 200 | intron16 | c.1837-8A>G (c.1836_1837insTTTCTAG) | p.A613FfsX25 Acceptor splice site error | Eeds et al., 2006 |
| 201 | 17 | c.1974delT | p.H659TfsX22 | Häberle et al., 2011 |
| 202 | Intron17 | c.1981+2T>G (c.1837_1981delExon17) | p.A613VfsX20 | Häberle et al., 2011 |
| 203 | 17 | C.2023delT | p.Cys675fs | Yang et al., 2017 |
| 204 | 18 | c.2115_2216ins35 | p.A717AfsX28 | Häberle et al., 2003 |
| 205 | 18 | c.2170_2173delGCTCinsCCA | p.A724PfsX27 | Rapp et al., 2001 |
| 206 | 18 | c.2173delC | p.L725WfsX19 | Häberle et al., 2011 |
| 207 | 19 | c.2227delC | p.L743X | Häberle et al., 2011 |
| 208 | 19 | c.2252delA | p.K751RfsX42 | Häberle et al., 2011 |
| 209 | 19 | c.2283_2287delTTTTGinsA | p.C761X | Häberle et al., 2011 |
| 210 | 19 | c.2338delC | p.R780VfsX13 | Eeds et al., 2006 |
| 211 | 20 | c.2404delG | p.G802VfsX19 | Häberle et al., 2011 |
| 212 | 20 | c.2494_2495insT | p.E832VfsX5 | Kurokawa et al., 2007 |
| 213 | 20 | c.2494delGinsAA | p.E832KfsX9 | Kurokawa et al., 2007 |
| 214 | 22 | c.2719_2721delGCAinsCC | p.A907PfsX25 | Häberle et al., 2011 |
| 215 | 22 | c.2797delT | p.L933X | Wakutani et al., 2004 |
| 216 | 22 | c.2809_2810delAT | p.I937PfsX5 | Häberle et al., 2011 |
| 217 | 23 | c.2876_2877delAT | p.Y959CfsX9 | Häberle et al., 2011 |
| 218 | 23 | c.2883_2895del13 | p.Y962SfsX11 | Eeds et al., 2006 |
| 219 | Intron 23 /Exon 24/Intron 24 | c.2895+429_c.2960-281del2559  (c.2896_2959delExon24) | p.E966AfsX27 | Häberle et al., 2011 |
| 220 | 25 | c.3044_3045insG | p.C1015WfsX4 | Eeds et al. 2006 |
| 221 | 26 | c.3185delA | p.N1062TfsX38 | Eeds et al. 2006 |
| 222 | Intron 26 | c.3337-1G>T (c.3337_3404delExon27) | p.N1113WfsX10 Acceptor splice site error | Häberle et al., 2011 |
| 223 | 27 | c.3341_3342insTTAAAATGA | p.E1114DX | Häberle et al., 2011 |
| 224 | 27 | c.3358_3359delAA | p.K1120VfsX25 | Summar et al., 1998 |
| 225 | 29 | c.3484delC | p.H1162TfsX5 | Wang et al., 2011; Häberle et al., 2011 |
| 226 | 29 | c.3485delA | p.H1162PfsX5 | Kretz et al., 2012 |
| 227 | 31 | c.3680_3681insTTTC | p.R1228FfsX24 | Häberle et al., 2011 |
| 228 | 32 | c.3869_3870insCC | p.E1290DfsX12 | Eeds et al., 2006 |
| 229 | 33 | c.3965_3966insCC | p.D1322AfsX5 | Eeds et al., 2006 |
| 230 | 33 | c.3969_3970insC | p.I1324HfsX5 | Kurokawa et al., 2007 |
| 231 | 33 | c.3969_3970insCC | p.I1324PfsX3 | Häberle et al., 2011 |
| 232 | Intron 34 | c.4102-239A>G (c.4101ins89) | p.Q1368SfsX17 | Häberle et al., 2011 |
| 233 | 33 | c.3972delT | p.L1325X | Rokicki et al., 2017 |
| 234 | 33 | c.3979delT | p.C1327VfsX21 | Ali et al., 2016 |
| 235 | 35 | c.4148delA | p.N1383MfsX44 | Häberle et al., 2011 |
| 236 | 37 | c.4397_4398insT | p.Q1468SfsX8 | Eeds et al., 2006 |
| 237 | Exon 37/Intron 37 | c.4404+1G>A (c.4275_4404delExon37) | p.L1426X | Häberle et al., 2011 |
| 238 | Intron 37 | c.4405-9T>G (c.4404ins8) | p.V1469IfsX2 | Häberle et al., 2011 |

**Table S4 In-frame deletions, insertions and splice site errors**

of the CPS1 gene

| **No** | **Exon** | **Nucleotide mutation** | **Protein** | **reference** |
| --- | --- | --- | --- | --- |
| 239 | Intron 2 | c.236+6T>C (mRNA change not determined) | Possible donor splice site error | Kurokawa et al., 2007 |
| 240 | 3 | c.306_311dupGAATGG | p.N103_G104insGN | Summar et al., 2011; Eeds et al., 2006 |
| 241 | Intron 4 | c.471+1G>A (c.382_271delExon4) | p.128V_157Kdel30 Donor splice site error | Häberle et al., 2011 |
| 242 | Intron 5 | c.529-3T>G (mRNA change not determined) | Acceptor splice site error | Eeds et al., 2006 |
| 243 | Intron7 | c.622-3C>G | Acceptor splice site error | This study |
| 243 | Intron 7 | c.622 -7A>G (c.621_622insTGGCAG) | p.K207_D208insWQ Acceptor splice site created within intron 6 | Häberle et al., 2011 |
| 244 | 7 | No DNA change identified (c.622_711delExon7) | p.D208_K236del30 | Häberle et al., 2011 |
| 245 | Intron 7 | c.711+1G>A (c.622_711delExon7) | p.D208_K236del30 Donor splice site error | Häberle et al., 2011 |
| 246 | 8-10 | c.711+686_1164+136del4260(c.712-1086delExons 8-10) | p.R238_E362del125 | Aoshima et al., 2001 |
|  | 8 | c.840G>C (c.832_840delGTCAGAAAG) | p.V278_K280delVRK | Hoshide et al., 1993 |
| 247 | Exon 9/Intron 9/Exon 10 | c.841-194_1086+77del1440(c.841_1086delExons9-10) | P.I281_E362del | Wang et al., 2011 |
| 248 | Exon 9/Intron 9/Exon 10/Intron 10/Exon 11 | c.841_212_c.1164+26del2395(c.841_1164delExons9-11) | P.I281_E388del108 | Wang et al., 2011 |
| 249 | Intron 10 | c.1087-1G>T (mRNA change not determined) | Acceptor splice site error | Eeds et al., 2006 |
| 250 | Intron 11 | c.1164+1G>A (c.1087_1164delExon11) | p.G363_E388del26 Donor splice site error | Häberle et al., 2011 |
| 251 | Intron 12 | c.1263+5G>C (c.1165-1263delExon12) | p.Y389_E421del33 Donor splice site error | Häberle et al., 2003 |
| 252 | 17 | c.1923_1925delTGA | p.D642del | Rokicki et al., 2017 |
| 253 | Intron 23 | c.2895+1G>A (c.2830_2895delExon23) | p.I944_Q965del22 Donor splice site error | Häberle et al., 2011 |
| 254 | 25 | c.2995_2997delAGT | p.S999del | Häberle et al., 2011 |
| 255 | 25 | c.3036_3038delGGT | p.V1011del | Häberle et al., 2003 |
| 256 | 25 | c.3037_3039delGTG | p.V1013del | Kretz et al., 2012 |
| 257 | 26 | c.3159_3161delCAT | p.I1054del | Häberle et al., 2011 |
| 258 | Intron 27 | c.3404+1G>C | Donor splice site error | Funghini et al., 2012 |
| 259 | Intron 28 | c.3405−8C>T | Acceptor splice site error | Funghini et al., 2012 |
| 260 | Intron 29 | c.3558+1G>C (c.3481_3558delExon29) | p.E1161_R1186del26 Donor splice site error | Häberle et al., 2003 |
| 261 | Intron 29 | c.3559-2A>G (c.3559_3666delExon30) | p.V1187_K1222del36 Acceptor splice site error | Häberle et al., 2011 |
| 262 | Intron 31 | c.3756+1G>A (c.3667-3756delExon31) | p.V1223_L1252del30 Donor splice site error | Häberle et al., 2011 |
| 263 | 34 | c.4088_4099del12 | p.L1363_I1366del | Häberle et al., 2011; Kretz et al., 2012 |
| 264 | Intron 34 | c.4101+2T>C (c.4003_4101delExon34+c.4101_4102ins42) | p.V1335-Q1367del33+ (p.Q1367_Q1368insASGLWLCACPWSYM) | Häberle et al., 2003; Klaus et al., 2009 |
